# Large-scale network organization in the avian forebrain: a connectivity matrix and theoretical analysis

**DOI:** 10.3389/fncom.2013.00089

**Published:** 2013-07-04

**Authors:** Murray Shanahan, Verner P. Bingman, Toru Shimizu, Martin Wild, Onur Güntürkün

**Affiliations:** ^1^Department of Computing, Imperial College LondonLondon, UK; ^2^Department of Psychology and J.P. Scott Center for Neuroscience, Mind and Behavior, Bowling Green State UniversityBowling Green, OH, USA; ^3^Department of Psychology, University of South FloridaTampa, FL, USA; ^4^Department of Anatomy with Radiology, Faculty of Medical and Health Sciences, University of AucklandAuckland, New Zealand; ^5^Biopsychologie, Fakultät für Psychologie, Ruhr-Universität BochumBochum, Germany

**Keywords:** brain connectivity, avain neuroanatomy, brain network analysis, pigeon forebrain, comparative neuroanatomy

## Abstract

Many species of birds, including pigeons, possess demonstrable cognitive capacities, and some are capable of cognitive feats matching those of apes. Since mammalian cortex is laminar while the avian telencephalon is nucleated, it is natural to ask whether the brains of these two cognitively capable taxa, despite their apparent anatomical dissimilarities, might exhibit common principles of organization on some level. Complementing recent investigations of macro-scale brain connectivity in mammals, including humans and macaques, we here present the first large-scale “wiring diagram” for the forebrain of a bird. Using graph theory, we show that the pigeon telencephalon is organized along similar lines to that of a mammal. Both are modular, small-world networks with a connective core of hub nodes that includes prefrontal-like and hippocampal structures. These hub nodes are, topologically speaking, the most central regions of the pigeon's brain, as well as being the most richly connected, implying a crucial role in information flow. Overall, our analysis suggests that indeed, despite the absence of cortical layers and close to 300 million years of separate evolution, the connectivity of the avian brain conforms to the same organizational principles as the mammalian brain.

## Introduction

Numerous recent studies have provided evidence for the cognitive prowess of birds. Corvids, such as rooks, crows, and jays, have proven especially fruitful subjects (Emery and Clayton, 2004), and have been shown to be capable of innovative tool manufacture (Weir et al., 2002), referential gesturing (Pika and Bugnyar, 2011), planning for future needs (Raby et al., 2007), mirror self-recognition (Prior et al., 2008), and causal reasoning (Taylor et al., 2012). Other species of birds, including pigeons (*Columba livia*, the focus of the present study), can also perform noteworthy feats of cognition, such as long-term recollection (Fagot and Cook, 2006), transitive inference (von Fersen et al., 1990), complex pattern recognition (Yamazaki et al., 2007), optimal choice (Herbransen and Schroeder, 2010), and numerical discrimination (Scarf et al., 2011). Although the cognitive accomplishments of birds are comparable to those of non-human mammals, their brains exhibit very different anatomical organization, as might be expected given that their most recent common ancestor was alive ~300 million years ago. Specifically, the pallium of a bird is nucleated and lacks the distinctive layers present in mammalian cortex (Jarvis et al., 2005).

Despite this fundamental difference, numerous studies have supplied evidence of underlying homologies (Reiner et al., 2004). A prominent example is the dorsal pallium, which constitutes the cortex in mammals, but is mostly organized as large unlaminated cell clusters in birds (Butler et al., 2011). However, certain cell groups within the unlaminated avian clusters are likely homologous with cortical laminae IV and V neurons (Dugas-Ford et al., 2012), and there is evidence of mammalian-like cortical lamination in the avian auditory forebrain (Wang et al., 2010). In general, the connectivity of the ascending sensory pathways, associative forebrain areas, and subpallial structures closely resembles the corresponding patterns of connectivity found in mammals (Kröner and Güntürkün, 1999; Reiner et al., 2005).

In short, there are marked parallels between the avian and mammalian forebrains, particularly at the level of connectivity, despite their radically different cytoarchitectural appearance, and it could be the case that these similarities in connectivity enable similar cognitive capacities. To analyse the overall connectivity of the avian telencephalon, we compiled a large-scale “wiring diagram” for the pigeon. To accomplish this, we drew on over four decades of pathway tracing studies to construct a connectivity matrix (a structural “connectome”) for the telencephalon of the pigeon. To the best of our knowledge, this is the first connectome to be published for the brain of any avian species—indeed the first for any non-mammalian vertebrate—and only the fourth for any vertebrate, following the cat, the macaque, and the human (Sporns, 2010). Using the mathematical tools of graph theory, we analysed the resulting matrix, producing a number of statistics and measures to facilitate comparison with similar studies on the three aforementioned mammalian species (Bullmore and Sporns, 2009). The analysis reveals that the forebrain of the pigeon is a disassortative, modular, small-world network with a connective core of hub nodes that bears close comparison to the cortices of the cat and macaque.

## Methods

**Figure 1:**
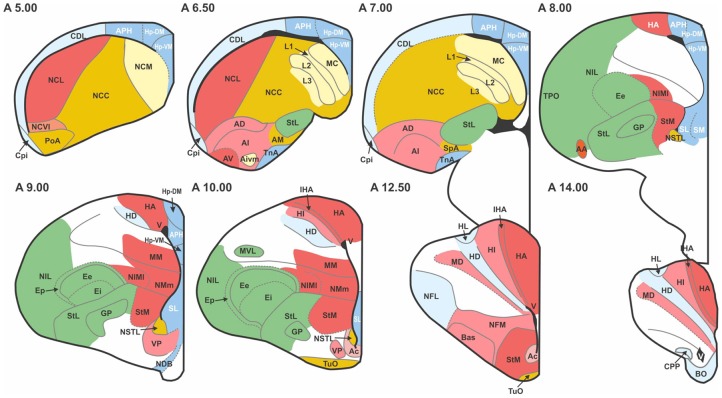
**Transverse sections through the pigeon telencephalon showing the locations of each of the 52 regions used in the study**. See Table 1 for abbreviations. Regions are colored according to their module and sub-module membership (see also Figure 4). Color codes: red, associative; blue, cortico-hippocampal; green, visual; brown, viscero-limbic; yellow, auditory. Regions colored white are excluded from the study. While the connections of these white regions have been explored, they have not been systematically clarified nor unequivocally confirmed. Black areas, such as the one labeled “V” at A14.00, are ventricles.

The pigeon (*Columba livia*) was chosen as a representative avian species, primarily because of the wealth of connectional information available. We considered all major structures of the pigeon telencephalon, including those within the pallium, striatum, pallidum, and septum, making no distinction between left and right hemispheres. Regions were delineated on the basis of standard cytoarchitectonic and neurochemical markers that have emerged from many decades of accumulated neuroanatomical research (Reiner et al., 2004). Altogether we defined 52 areas for which there is strong neuroanatomical evidence of differentiation (Figure 1; Table 1). We then carried out a comprehensive survey of tract tracing studies of the pigeon forebrain to establish all known connections among pairs of regions under consideration. Each of the selected 52 regions has been the target of at least one tracer study. As such, there is an opportunity to discover every potential pathway between the identified regions. With the exception of the prepiriform cortex (CPP), subpallial amygdala (SPA), and olfactory tubercle (TUO), for which only anterograde tracing evidence is available, all other delineated areas have been investigated using both retrograde and anterograde tracers. Table 2 summarizes our database.

**Table 1:** **Regions included in the study and their abbreviations**.

AA (Arcopallium anterior)	Hp-VM (Ventromedial nucleus of hippocampus)
Ac (N. accumbens)	MC (Mesopallium caudale)
AD (Arcopallium dorsale)	MD (Mesopallium dorsale)
AI (Arcopallium intermedium)	MM (Mesopallium mediale)
AIvm (Arcopallium intermedium pars ventromedialis)	MVL (Mesopallium ventrolaterale)
AM (Arcopallium mediale)	NCC (Central caudal nidopallium)
APH (Area parahippocampalis)	NCL (Nidopallium caudolaterale)
AV (Arcopallium ventrale)	NCM (Nidopallium caudomediale)
Bas (N. basalis prosencephali)	NCVl (Nidopallium caudoventrale pars lateralis)
BO (Bulbus olfactorius)	NDB (N. diagonalis Broca)
CDL (Area corticoidea dorsolateralis)	NFL (Nidopallium frontolaterale)
CPi (Cortex piriformis)	NFM (Nidopallium frontomediale)
CPP (Cortex prepiriformis)	NIMl (Nidopallium intermedium mediale pars lateralis)
Ei (Entopallium internum)	NMm (Nidopallium mediale pars medialis)
Ee (Entopallium externum)	NIL (Nidopallium intermedium laterale)
Ep (Entopallial belt)	NSTL (Bed nucleus of the stria terminalis)
Field L1	PoA (N. posterioris amygdalopallii)
Field L2	SL (Septum laterale)
Field L3	SM (Septum mediale)
GP (Globus pallidus)	SpA (Area subpallialis amygdalae)
HA (Hyperpallium apicale)	StL (Striatum laterale)
IHA (N. interstitialis hyperpallii apicalis)	StM (Striatum mediale)
HI (Hyperpallium intercalatum)	TnA (N. taeniae amygdalae)
HD (Hyperpallium densocellulare)	TPO (Area temporoparietalis)
HL (Hyperpallium laterale)	TuO (Tuberculum olfactorium)
Hp-DM (Dorsomedial nucleus of the hippocampus)	VP (Ventral pallidum)

**Table 2:** **Directional connections of all structures**.

AA	→ AD: 29, 34; ← AD: 29; → AI: 19; ← AI: 34; ← Ep: 12, 19; ← Field L1: 19; ← Field L3: 19; → HI: 7; → HD: 7; → MD: 7; → NCL: 19; ← NCL: 19, 34; ← NCM: 34; → NFM: 19; → StL: 19, 29; → StM: 19, 29; → TPO: 3, 34; → TuO: 29; → VP: 34
Ac	← AD: 13, 29; ← AI: 13, 29; ← APH: 13, 29; ← CPi: 9, 13, 29; ← Hp-DM: 22, 13; ← MM: 13; ← NCC: 13; ← NCL: 13, 19; ← NFM: 13; ← NMm: 13; ← NSTL: 1, 13; ← PoA: 1, 13, 29; → VP: 13, 29
AD	→ AI: 1, 19, 29; ← AI: 19, 34; → CPi: 23; ← Ep: 12, 19; ← Field L1: 19; ← Field L3: 19; → HI: 7; ← HI: 7; → HD: 7; ← HD: 28; → MD: 4, 5; ← MD: 4, 5, 28; → MM: 5; ← MM: 5; → NCL: 19; ← NCL: 1, 19, 34; ← NFL: 19; → NFM: 19; ← NMm: 19; ← NSTL: 1; → SL: 2; ← SL: 2; ← SM: 2; → StL: 1, 19, 29; ← StM: 1, 19, 29; → TPO: 3, 34; → TuO: 29; → VP: 22, 29;
AI	→ AM: 4, 19; → APH: 10; ← APH: 10; ← Ei: 12; ← Ee: 12; ← Ep: 12, 19; ← Field L1: 19; ← Field L3: 19; ← HA: 19, 28; → HI: 7, 28; ← HI: 28; → HD: 7; ← HD: 28; → MC: 5, 19; → MD: 5; ← MD: 5, 28; → MM: 5; ← MM: 5, 7; → NCC: 4; → NCL: 19, 21, 24; ← NCL: 19, 21, 24, 34; ← NCM: 1, 19; ← NFL: 19; → NFM: 19, 27, 31, 32; ← NFM: 19, 31, 32; ← NIL: 19; → NIMl: 19; ← NIMl: 19; → NMm: 19; ← NMm: 19; → SL: 2; → StL: 19, 29; → StM: 19, 29; → TPO: 3; ← TPO: 34; → TuO: 29; → VP: 29, 34
AIvm	→ Field L1: 33; → Field L3: 33; → MC: 33; ← NCL: 19, 33
AM	← NCC: 4; → NCL: 4, 19; → NSTL: 1; ← NSTL: 1; ← PoA: 1; → SL: 2; ← SL: 2; → StM: 13, 29; ← TnA: 4
APH	→ AV: 4, 19; ← AV: 10; → CDL: 2, 3, 10, 17, 19; ← CDL: 2, 3, 8, 10; ← CPi: 2; ← HA: 19, 28; → HD: 2; ← HD: 2, 10, 28; → HL: 2; ← HL: 2, 10; → Hp-DM: 2, 6, 10, 14; ← Hp-DM: 2, 6, 10, 14; → Hp-VM: 2, 6, 14; ← Hp-VM: 2, 6, 14; → NDB: 2, 10; ← NDB: 2, 8, 10, 18; ← NFL: 2; → SL: 2, 10, 17; ← SL: 2; → SM: 2, 10, 17; ← SM: 2, 8, 10; ← SpA: 10; → TnA: 2, 17; ← TnA: 2
AV	← BO: 23; → MD: 4, 5; ← MD: 4, 5; ← NCL: 19; → NIMl: 19; → NMm: 19; → StL: 29; → StM: 19, 29
Bas	→ NFM: 27, 31, 32; ← NFM: 27, 31, 32
BO	→ CDL: 23, 25; → CPi: 9, 23, 25, 26; → CPP: 23, 25; → SM: 23; → TnA: 23, 25
CDL	→ CPi: 3, 23; ← CPi: 3, 23; → HD: 3; ← HD: 3, 28; → HL: 3; ← HL: 3; → Hp-DM: 2, 3, 6, 10; ← Hp-DM: 2, 3, 6, 10; → Hp-VM: 3, 6; ← Hp-VM: 3, 6; → MD: 5; ← MD: 3, 5; → NCVl: 3; ← NCVl: 3; → NDB: 3; → NFL: 3; ← NFL: 3; → NIL: 3; ← NIL: 3; → PoA: 1, 3; ← PoA: 1, 3; → SL: 3; → StL: 3, 19, 29; → TPO: 3; ← TPO: 3;
CPi	← CPP: 9, 23; ← HD: 9, 23, 28; ← HL: 23; ← NFL: 23; ← NSTL: 1. 9, 23, 29; ← PoA: 1, 23, 29
Ee	← MVL: 5, 20, 24
Ei	→ Ep: 12, 16, 20, 24; Ei → MVL: 5, 12, 20, 24; ← MVL: 5, 12, 20, 24; ← NFL: 20, 29; → NIL: 12, 20; → StL: 12, 20
Ep	→ NCL: 19; ← NCL: 12, 19; → NFL: 12, 20, 29; → NIL: 12, 20; → TPO: 20
L1	← Field L2: 33; ← Field L3: 33; → MC: 5, 24; ←MC: 5; →NCL: 19, 33; ← NCL: 19, 33;
L2	→ Field L3: 33; ← Field L3: 33; → MC: 33; ← MC: 5, 33;
L3	→ MC: 5, 33; ← MC: 5, 33; ← NCL: 19
GP	← StL: 15, 22, 29
HA	← HD: 19, 28; ← HI: 19, 28; → HL: 28; ← HL: 28; → Hp-DM: 10; ← Hp-DM: 19; ← IHA: 19, 28, 30; → MM: 5, 19; → NCL: 19, 21, 28; ← NCL: 19; → NFL: 19, 28; → NIMl: 19, 28; ← NIMl: 11, 19; → NMm: 19, 28, 30; → StL: 28, 29; → StM: 28, 29; → TPO: 5, 19, 28
HD	→ Hp-DM: 2, 10; ← Hp-DM: 2; → MM: 5; → NCL: 19, 21, 28; → NFL: 28; → PoA: 1; ← PoA: 1; → StL: 28, 29; → StM: 19, 28, 29
HL	→ Hp-DM: 2, 10; ← Hp-DM: 2; → NCL: 19, 21; ← NCL: 19; → NFL: 28;
Hp-DM	→ Hp-VM: 2, 6, 17; ← Hp-VM: 2, 6; → NDB: 6, 10, 14, 17; ← NDB: 6, 10, 14; → NSTL: 1, 6, 17; ← PoA: 1, 2; → SL: 2, 6, 10, 17; ← SL: 2, 6; → SM: 2, 6, 14, 17; ← SM: 2, 6; ← SpA: 2; → TnA: 2, 17; ← TnA: 10
Hp-VM	→ NDB: 6, 17; ← PoA: 6; → SL: 2, 6, 10, 17; ← SL: 2, 6; ← SM: 2, 6; → TnA: 6; ← TnA: 6
MC	→ MM: 5; → NCM: 5; ← NCM: 5; → NIMl: 5, 19; ← NIMl: 5, 19, 24; → NMm: 24
MD	→ NCC: 4, 24, 28; ← NCC: 4, 24; → NCL: 5, 19, 21, 24, 28; ← NCL: 5, 24; → NCM: 4, 24, 28; → NFL: 28; → NFM: 5; ← NFM: 5; → NMm: 4, 5, 19, 28; → PoA: 4, 5; → StM: 5, 19, 24, 28, 29; → TPO: 3, 5, 28
MM	→ NCL: 5, 19; ← NCL: 19, 24; → NMm: 5, 19; ← NMm: 5; → StL: 5, 24, 29; → StM: 5, 13, 19, 24, 29
NCC	← NMm: 4; → NSTL: 1, 4; ← NSTL: 1; ← PoA: 1, 4
NCL	→ NCM: 1; ← NCM: 1, 19; ← NFL: 19; ← NFM: 19, 24, 27, 31, 32; → NIMl: 19; ← NIMl: 19, 24; → NMm: 19, 24; ← NMm: 19; → NSTL: 1, 19; ← PoA: 1, 19, 21; → StL: 19, 29; → StM: 19, 29; ← TnA: 19, 21
NCM	← NIMl: 19
NCVl	→ NSTL: 1; ← NSTL: 1; → PoA: 1; ← PoA: 1
NDB	→ SL: 2; → SM: 2; ← SM: 2
NFL	→ PoA: 1; ← PoA: 1; → StL: 20, 29; → StM: 29; → TPO: 3
NFM	← PoA: 1
NIL	→ StL: 20, 29; → TPO: 3
NIMl	→ StL: 19
NMm	→ StM: 13, 19, 29
NSTL	→ PoA: 1; ← PoA: 29; → SL: 1, 2; ← SL: 1, 2; → SpA: 1; ← SpA: 1; → TnA: 1; ← TnA: 29; → TuO: 1; ← TuO: 1
PoA	→ SL: 1, 2; → SpA: 1; → StL: 1; → TuO: 1, 29; ← TuO: 1
SL	→ SM: 2; ← SM: 2; ← TnA: 2, 25; ← TuO: 2; → VP: 2; ← VP: 2
SM	→ TuO: 2; ← TuO: 2; → VP: 2; ← VP: 2
StL	← TPO: 3, 19, 20, 29; → VP: 15, 29
StM	→ VP: 22, 29

*Numbers denote citations (full citations in reference list). BDA, biotynilated dextrane amine; CTb, Cholera toxin subunit b; deg, degeneration fiber tracing (Fink-Heimer); ^3^H-leucine, autoradiography using ^3^H leucine; ^3^H-proline, autoradiography using ^3^H proline; HRP, horseradish peroxidase; Pha-L, Phaseolus vulgaris leucoagglutinin; RITC, Rhodamine isothiocyanate; WGA-HRP, what germ aggl. HRP. Citations: 1, Atoji et al., 2006 [BDA, CTb]; 2, Atoji and Wild, 2004 [BDA, CTb]; 3, Atoji and Wild, 2005 [BDA, CTb]; 4, Atoji and Wild, 2009 [BDA, CTb]; 5, Atoji and Wild, 2012 [BDA, CTb]; 6, Atoji et al., 2002 [BDA, CTb]; 7, Bagnoli and Burkhalter, 1983 [HRP]; 8, Benowitz and Karten, 1976 [HRP, deg.]; 9, Bingman et al., 1994 [Fast Blue, WGA-HRP]; 10, Casini et al., 1986 [WGA-HRP, ^3^H-proline]; 11, Funke, 1989 [HRP]; 12, Husband and Shimizu, 1999 [BDA, Pha-L]; 13, Husband and Shimizu, 2011 [BDA, CTb]; 14, Kahn et al., 2003 [BDA, CTb]; 15, Karten and Dubbeldam, 1973 [deg.]; 16, Karten and Hodos, 1970 [deg.]; 17 Krayniak and Siegel, 1978a [^3^Hleucine]; 18, Krayniak and Siegel, 1978b [^3^Hleucine]; 19, Kröner and Güntürkün, 1999 [BDA, CTb]; 20, Krützfeldt and Wild, 2005 [BDA, CTb]; 21, Leutgeb et al., 1996 [Fast Blue, CTb]; 22, Medina and Reiner, 1997 [BDA]; 23, Patzke et al., 2011 [CTb, BDA]; 24, Rehkämper and Zilles, 1991 [^3^Hleucine, WGA-HRP, HRP]; 25, Reiner and Karten, 1985 [^3^Hproline, [^3^Hleucine]; 26, Rieke and Wenzel, 1978 [deg.]; 27, Schall et al., 1986 [HRP]; 28, Shimizu et al., 1995 [Pha-L, CTb]; 29, Veenman et al., 1995 [Fluorogold, Fast Blue, RITC, WGA-HRP, BDA]; 30, Wild, 1987 [WGA-HRP]; 31, Wild et al., 1985a [^3^Hproline, ^3^Hleucine, WGA-HRP]; 32, Wild et al., 1985b [^3^Hproline, ^3^Hleucine, WGA-HRP]; 33, Wild et al., 1993 [BDA, CTb-HRP, WGA-HRP, CTb, CTb-Gold, Pha-L, RITC/Fluoresc. spheres]; 34, Zeier and Karten, 1971[deg.]*.

**Figure 2:**
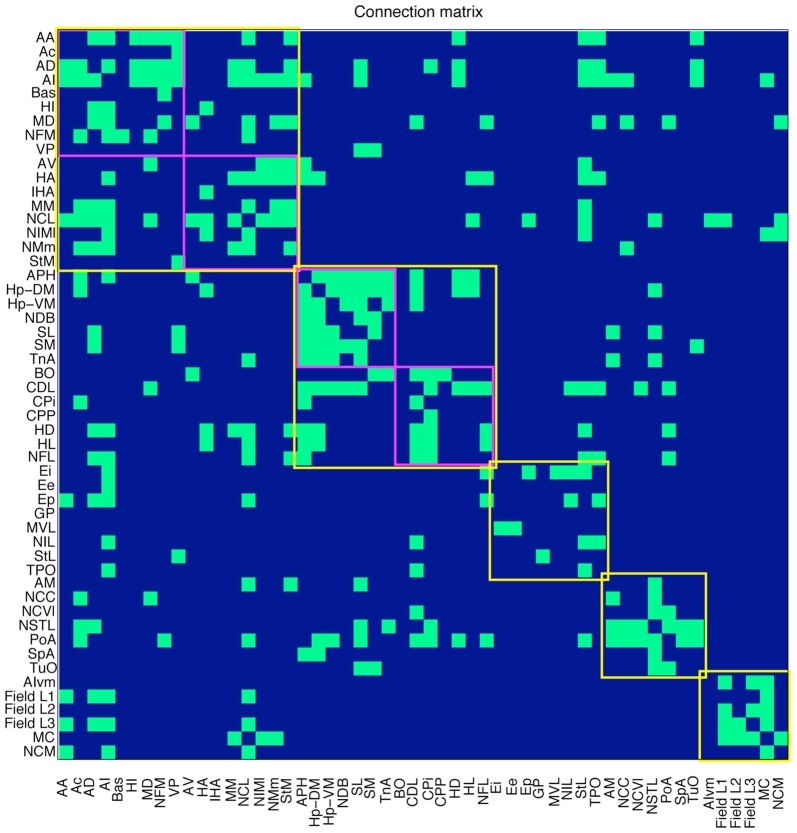
**Connections in the pigeon telencephalon**. A green cell in row *i*, column *j* indicates that a connection exists from region *i* to region *j*. Top-level modules are outlined in yellow. Sub-modules are outlined in magenta. See Table 1 for abbreviations.

Based on this published evidence, each cell in the 52 by 52 matrix was assigned a value of 0 or 1, where 0 indicates that no evidence for the existence of the relevant pathway has been reported in the literature, and 1 indicates that there is experimental proof for the existence of a connection. The resulting sparse matrix corresponds to a directed graph, in which each node represents an anatomical region and each arc represents a connection. The matrix, which contains 344 connections, is therefore a distillation of the complete connectome of a single hemisphere of the pigeon forebrain (Figure 2).

A number of statistics were computed using Matlab functions from the Brain Connectivity Toolbox (Rubinov and Sporns, 2010), including (directed) clustering coefficient (Fagiolo, 2007) and distance matrix (both used to compute small-world index), modularity (Leicht and Newman, 2008), and betweenness centrality (Freeman, 1977). The standard definition of participation coefficient (Guimerá et al., 2007) was adapted for the directed case.

### Small-world indices

Two small-world indices, σ and σ_io_, were calculated. We have

$$\sigma =\frac{\gamma /\gamma_{r}}{\lambda /\lambda_{r}}$$

where γ is the (directed) clustering coefficient of the network (Fagiolo, 2007), and λ is its mean path length. γ_*r*_ and λ_*r*_ are, respectively, the expected clustering coefficient and mean path length of a random network with the same number of nodes *n* and average node degree *k*. These values were estimated by generating 200 random networks and calculating their average clustering coefficients and path lengths [yielding values that agree with analytical expressions for directed graphs, namely γ_*r*_ = *k/n* and λ_*r*_ = ln(*n*)/ln(*k*) (Watts and Strogatz, 1998)], as well as the corresponding z-scores. Clustering coefficients and distance matrices (used to calculate mean path length) were computed using Matlab functions from the Brain Connectivity Toolbox (Rubinov and Sporns, 2010).

Similarly, we have

$$\sigma_{\text{io}}=\frac{\gamma /\gamma_{\text{rio}}}{\lambda /\lambda_{\text{rio}}}$$

where γ_rio_ and λ_rio_ are the expected clustering coefficient and mean path length of a random network with the same degree sequence. Two networks A and B have the same degree sequence if there is a one-to-one mapping from every node in A to a node in B with the same in-degree and out-degree. Again, these values were estimated by generating 200 random networks with the requisite degree sequence and calculating their average clustering coefficients and path lengths, as well as corresponding z-scores. The comparative values of σ and σ_io_ for macaque and cat cortex were obtained from statistics reported by Sporns and Zwi (2004).

### Assortativity

The assortativity coefficient *r* for a directed network with a set *L* of edges, where *l* is the cardinality of *L*, is defined as

$$r=\frac{\frac{1}{l}\sum_{(i,\;j)\in L}k_{i}^{\text{out}}k_{j}^{\text{in}}-{\left(\frac{1}{l}\sum_{(i,\;j)\in L}0.5\text{​}\left(k_{i}^{\text{out}}+k_{j}^{\text{in}}\right)\right)}^{2}}{\frac{1}{l}\text{​}\sum_{(i,\;j)\in L}0.5\text{​}\left(\text{​}{\left(k_{i}^{\text{out}}\right)}^{2}\text{​}+\text{​}{\left(k_{j}^{\text{in}}\right)}^{2}\right)-{\left(\text{​}\frac{1}{l}\text{​}\sum_{(i,\;j)\in L}0.5\text{​}\left(k_{i}^{\text{out}}+k_{j}^{\text{in}}\right)\text{​}\right)}^{2}}$$

where *k*^in^_*i*_ and *k*^out^_*i*_ are the in-degree and out-degree of node *i*, respectively (Newman, 2003; Fagiolo, 2007).

### Motifs

An *n*-motif is a connected, directed graphs comprising exactly *n* nodes (Milo et al., 2002). There are 13 distinct 3-motifs and 199 possible 4-motifs. Using the Brain Connectivity Toolbox, the number of occurrences of every distinct 3-motif and 4-motif in the pigeon connectome was computed. The *z*-score for motif *i* is then

$$z_{i}=\frac{Mo_{i}-\mu_{i}}{{\text{sd}}_{i}}$$

where *Mo*_*i*_ is the number of occurrences of motif *i*, μ_*i*_ is the expected number of occurrences of motif *i* in a random network with identical degree sequence, and sd_*i*_ is the corresponding standard deviation. Expected values and standard deviations were estimated by generating 200 random networks.

### Modularity

The modularity analysis was based on the measure *Q* which assesses the modularity of a given partitioning of a network into *m* communities (modules) and is defined as (Leicht and Newman, 2008)

$$Q=\frac{1}{2m}\sum_{i,\;j}\left(A_{ij}-\frac{k_{i}^{\text{in}}k_{j}^{\text{out}}}{2m}\right)\delta_{c_{i}c_{j}}$$

where *A*_*ij*_ is the value of the connection from node *j* to node *i*, *k*^in^_*i*_ and *k*^out^_*i*_ are the in-degree and out-degree of node *i* respectively, *c*_*i*_ is the community (module) number of node *i*, and

$$\delta_{xy}=\{\begin{array}{l} 1\text{ if }x=y\\ 0\text{ otherwise}. \end{array}$$

The aim is to find a partitioning of the network that maximizes *Q*. In general this is computationally intractable, but stochastic methods can be used that are effective at finding partitions with high *Q*. We used the Matlab function from the Brain Connectivity Toolbox to do this, running it 100 times and selecting the partitioning that yielded the highest value for *Q*.

### Hubs

Hub designations were based on betweenness centrality (Guimerá et al., 2007), again using the Brain Connectivity Toolbox. The betweenness centrality BC of a node *h* is defined as

$$\text{BC}(h)=\sum_{i,\;j,\;i\;\neq \;j\;\neq \;h}\frac{g_{ij}(h)}{g_{ij}}$$

where *g*_*ij*_ is the number of shortest paths from node *j* to node *i* and *g*_*ij*_ (*h*) is the number of shortest paths from *j* to *i* that pass through *h*. Further designation of a node *i* as a connector hub depended on its participation coefficient (Guimerá et al., 2007). For a directed network, given a partitioning into communities (modules), this is defined as

$$P(i)=1-\frac{1}{2}\left(\sum_{c}{\left(\frac{k_{i}^{\text{in}}(c)}{k_{i}^{\text{in}}}\right)}^{2}+\sum_{c}{\left(\frac{k_{i}^{\text{out}}(c)}{k_{i}^{\text{out}}}\right)}^{2}\right)$$

where *k*^in^_*i*_ (*c*) is the number of incoming connections to node *i* from community *c* and *k*^out^_*i*_ (*c*) is the number of outgoing connections from node *i* to community *c*. (See Table 4, right.).

### K-core decomposition

The process of *k*-core decomposition successively removes from the network nodes that have *i* or fewer connections, beginning with *i* = 1 and increasing *i* until the network is fully eroded. The order in which the nodes are removed reveals a nested series of *k*-cores and sub-shells, where the innermost sub-shell contains the nodes that are most resistant to the erosion process. More precisely, the *i*^th^ *k*-core of a network is the set of nodes in the largest sub-graph of the network that contains only nodes of degree *i* or above. To obtain the *i* + 1^th^ *k*-core of a network from the *i*^th^ *k*-core entails peeling away a series of sub-shells. The first sub-shell of the *i*^th^ *k*-core is the set of nodes in the corresponding sub-graph with degree *i*. After removing these nodes from the sub-graph corresponding to the *i*^th^ *k*-core, some of the remaining nodes may have been reduced to degree *i*, so these now need to be removed. The set of such nodes, if it is non-empty, constitutes the second sub-shell. In general, the *j*^th^ sub-shell of the *i*^th^ *k*-core is the set of nodes with degree *i* remaining after sub-shells 1 to *j*–1 and their associated edges have been removed. The *i*^th^ *k*-core is therefore the union of its sub-shells and the *i* + 1^th^ *k*-core. [The formally defined concept of a *k*-core should not be confused with the wider notion of a connective core (Shanahan, 2012), although the former can be used to help designate the latter.] The algorithm used here was based on that of Modha and Singh (2010) (see their Supporting Information, pp. 4–5), and is given in Appendix.

### Rich club analysis

Given a ranking of the nodes in a network, the *rich club coefficient* for rank *k* is defined as

$$\phi (k)=\frac{2E_{k}}{N_{k}\left(N_{k}\phi 1\right)}$$

where *E*_*k*_ is the number of edges between nodes of rank greater than or equal to *k*, and *N*_*k*_ is the number of such nodes (Zhou and Mondragón, 2004). Nodes are ranked according to total degree, with the highest ranking node having the highest degree. Relative rankings of nodes of equal total degree can be assigned to maximize **ϕ(*k*)**. The *normalized rich club* coefficient for rank *k* is **ϕ(*k*)**/**ϕ**^rand^ **(*k*)**, where **ϕ**^rand^ **(*k*)** is the expected rich club coefficient of a randomly generated network with the same degree sequence, which was here estimated by constructing 200 such networks. The set of nodes of rank *k* and above is considered a rich club if every such node has a normalized rich club coefficient greater than one.

### Knotty-centrality

The *knotty-centrality* of a subset *S* of the nodes in a network is defined as

$$\text{KC}(S)=\frac{E_{S}}{N_{S}(N_{S}-1)}\sum_{i\;\in \;S}\text{bc}(i)$$

where *E*_*S*_ is the number of edges between nodes in *S*, and *N*_*S*_ is the number of nodes in *S* (Shanahan and Wildie, 2012). bc(*i*) is the betweenness centrality of node *i* normalized with respect to the whole network, such that

$$\text{bc}(i)=\frac{\text{BC}(i)}{\sum_{j\;\in \;G}\text{BC}(j)}$$

where *G* is the set of all nodes in the network and BC(*i*) is the betweenness centrality of node *i* as defined above. A subset *S* of the nodes in a network is a *knotty center* of that network if there is no *S*′ such that KC(*S*′) < KC(*S*). The knotty center of the graph was found using the algorithm of Shanahan and Wildie (2012).

## Results

The connectivity matrix resulting from our meta-analysis is given in Table 2. This was analysed using several mathematical measures from network theory as described in the Methods. First, the overall network was found to exhibit small-world properties. It has a clustering coefficient of γ = 0.3647, which is significantly higher than the average clustering coefficient for both (a) random networks with the same number of nodes and edges (γ_*r*_ ≈ 0.2591, z-score = 25.90), and (b) random networks that also have the same degree sequence (γ_rio_ ≈ 0.2514, z-score = 7.42). However, despite the high clustering coefficient, the pigeon telencephalon retains a low mean path length of λ = 2.3961. Although this is significantly higher than the average path length of λ_*r*_ ≈ 1.7629 for a random network with the same number of nodes and edges (z-score > 100), it is comparable to the average of λ_rio_ ≈ 2.3133 for a random network with the same degree sequence (z-score = 2.84). These statistics yield an insignificant small-world index of σ = 1.0356 when normalized to a random network. But when normalized while preserving degree sequence, the statistics yield a small-world index for the pigeon telencephalon of σ_io_ = 1.4004, which falls between the corresponding indices for macaque cortex (σ_io_ = 1.7050) and cat cortex (σ_io_ = 1.3027) (Sporns and Zwi, 2004).

The network's assortativity coefficient, which quantifies the extent to which nodes connect to other nodes with similar degree (Newman, 2002), is *r* = −0.1143. A network with low assortativity is thought to be less “robust” than a network with positive assortativity, because damage to a high degree node will tend to have a more systemic effect on connectivity (Newman, 2002). However, the significance of low assortativity in brain networks remains unclear. For macaque cortex, *r* = −0.0066 for the 71-node dataset of Young (1993), and for cat cortex *r* = −0.0394 for the 52-node dataset of Scannell et al. (1999). For human brain structural connectivity both high and low values have been found in different studies (Hagmann et al., 2008; van den Heuvel and Sporns, 2011).

**Figure 3:**
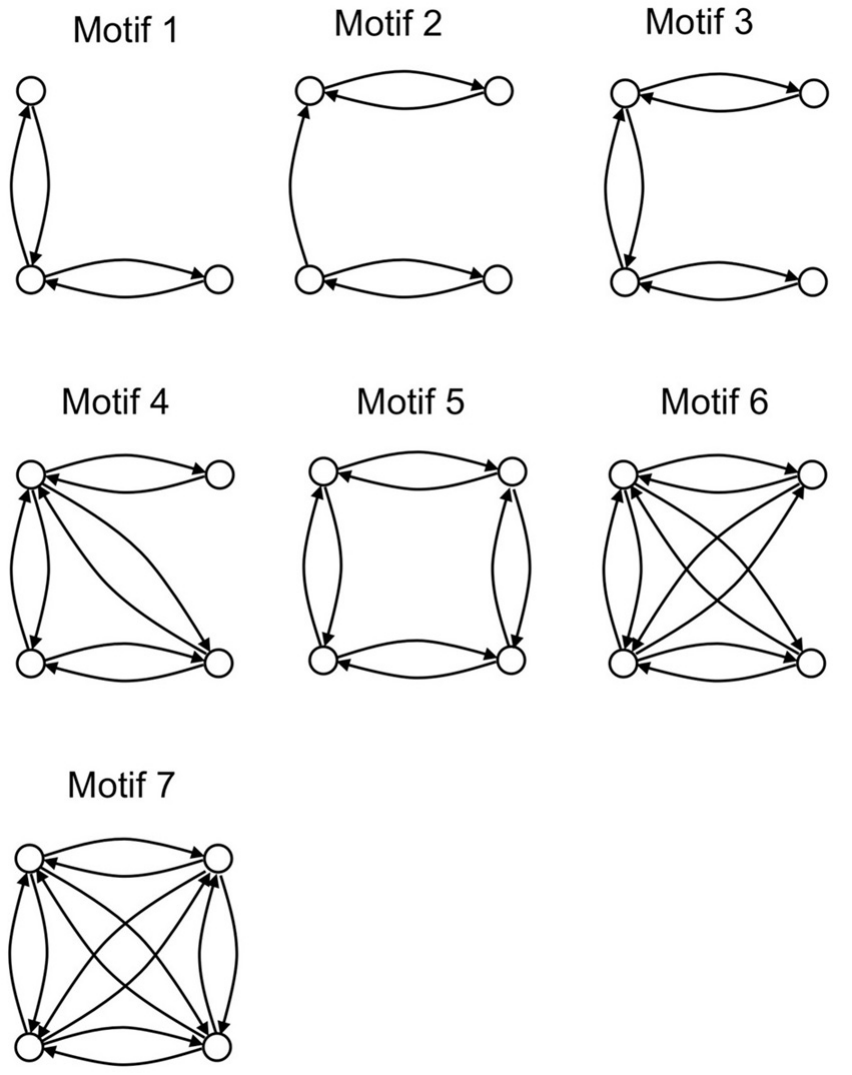
**The seven structural motifs that occur with highest z-scores**. Circles denote nodes (brain regions) and arrows denote directed arcs (connections).

An analysis of the local connectivity of the network yields seven structural motifs that occur with high z-scores (>12) with respect to 200 randomly generated networks with the same degree sequence (Figure 3). Three of these pigeon forebrain motifs (numbers 1, 3, and 7) are included in a set of five motifs that were reported to occur with high z-scores in both macaque and cat cortex (Sporns and Kötter, 2004). According to Sporns and Kötter, an abundance of motif 1 and its 4-node extensions (such as motifs 3, 4, and 7) supports a blend of integrated and segregated dynamics, because these motifs contain chains of reciprocally connected nodes (promoting integration), whose end nodes are disconnected (promoting segregation) (Sporns and Kötter, 2004).

Also in line with results for macaque and cat cortex, the number of structural motifs (of size 3 and 4) is less for the pigeon network than for an equivalent random network with identical degree sequence. Only 2328 3-node motifs occur in the pigeon network compared to an average of 3405 in 200 randomly generated equivalent networks, while only 22,114 4-node motifs occur compared to an average of 37,156 in 200 randomly generated equivalents. As with the cat and macaque, the structural motifs that are more prevalent than expected are highly connected (Figure 3), and therefore allow for a larger than expected number of functional configurations (Sporns and Kötter, 2004).

**Figure 4:**
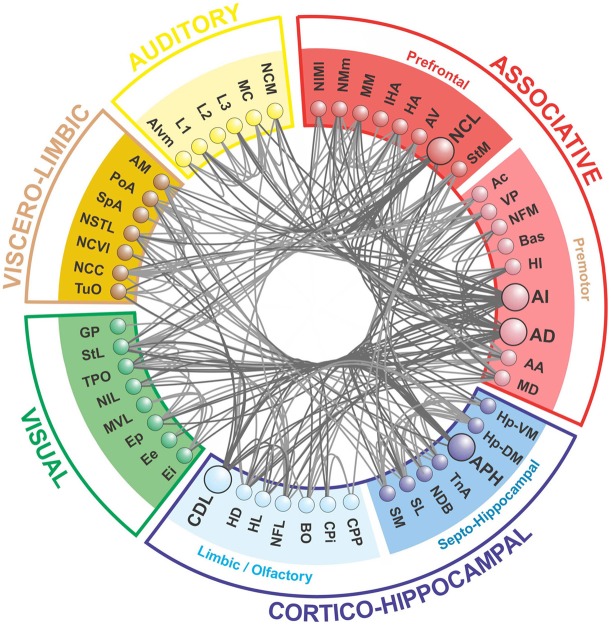
**The telencephalic connectome of the pigeon forebrain**. Network analysis reveals five top-level modules. The associative and cortico-hippocampal modules can be further decomposed. Connections to and from hub nodes are shown in a slightly darker color. See Table 1 for abbreviations.

Our modularity analysis revealed a partitioning of the network into five distinct subsets of regions (modules), the members of which are more densely connected to each other than to regions in other subsets (Figure 4). Repeated application of the iterative procedure described in the Methods (with newly generated seeds for the random number generator) always yielded the same partitioning of the nodes into two large modules (with *Q* = 0.2796) and seven sub-modules. However, subsequent hand-tuning of this partitioning yielded a five-module partitioning with an increased value of *Q* = 0.3020 that also made more neuroanatomical sense. [As Good et al. have demonstrated (Good et al., 2010), there are likely to be multiple high-*Q* partitioning's of any given network, which licenses a degree of subjective selectivity.] A further level of analysis revealed the finer modular structure of the two largest top-level modules, yielding *Q* = 0.1309 for the “associative module” and *Q* = 0.1932 for the “cortico-hippocampal” module.

What we call the “associative module” is the largest of the five top-level modules in terms of node membership. It includes the central associative structure of the avian brain (NCL; Nidopallium caudolaterale), diverse premotor and motor areas (AI, AD; Arcopallium intermedium, Arcopallium dorsale, respectively), as well as several primary sensory and associative structures of the visual, auditory, trigeminal, and somatospinal systems. Recursively applying the modularity analysis reveals a further level of hierarchy comprising a “prefrontal” sub-module and a “premotor” sub-module.

What we call the “cortico-hippocampal” module is the second largest, and includes major areas of the hippocampal formation, including APH (Area parahippocampalis), as well as structures such as CDL (Area corticoidea dorsolateralis), which are gateways between sensory and limbic areas and the hippocampus. It also contains limbic components, such as the septum and parts of the amygdala, which directly connect to the hippocampal formation. In addition, olfactory structures are part of this module. Again, a further level of hierarchy is revealed by recursive modularity analysis, which partitions this module into a “septo-hippocampal” sub-module and a “limbic/olfactory” sub-module.

**Figure 5:**
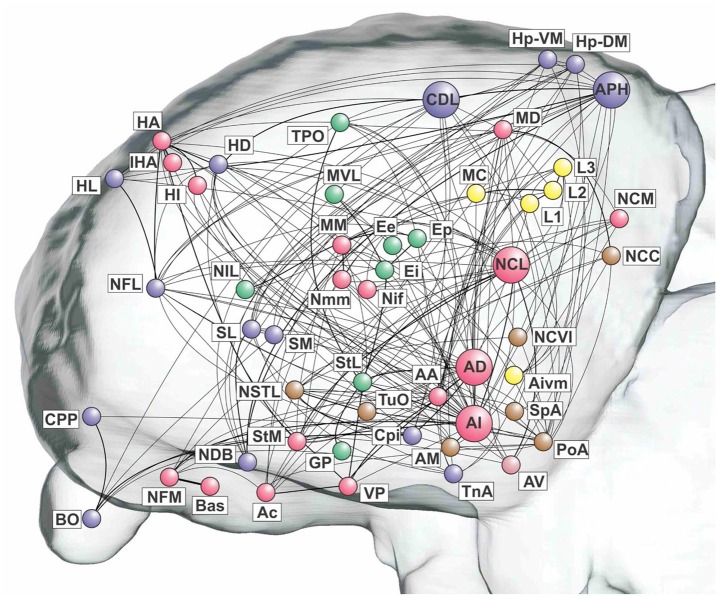
**Pathways of the pigeon forebrain in anatomical co-ordinates (sagittal view)**. Nodes are colored according to top-level module membership. Note that the modules are spatially distributed rather than localized. See Table 1 for abbreviations. See also Figure 1 for color codes: red, associative; blue, cortico-hippocampal; green, visual; brown, viscero-limbic; yellow, auditory.

**Figure 6:**
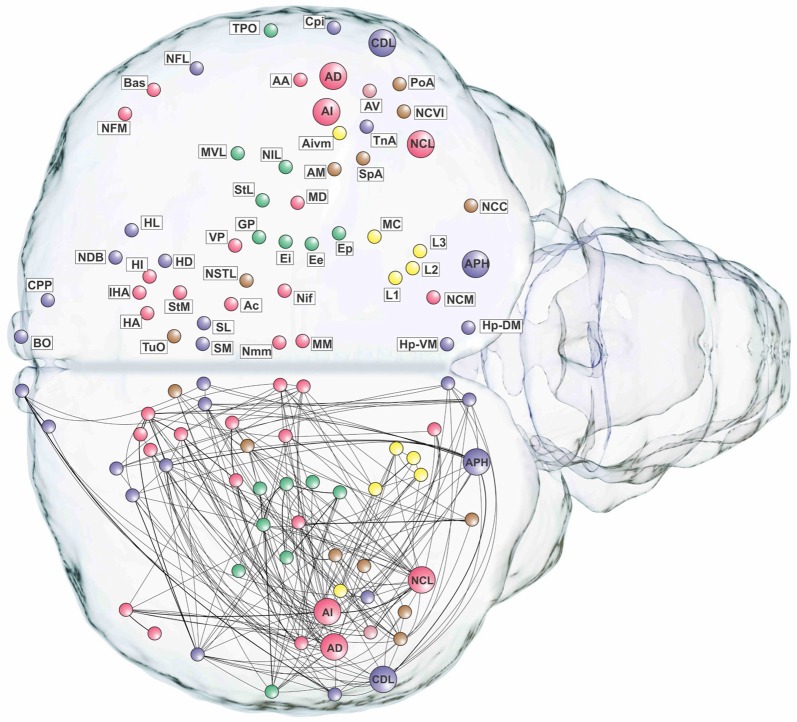
**Pathways of the pigeon forebrain in anatomical co-ordinates (horizontal view)**. Nodes are colored according to top-level module membership (Figure 4). Note the spatial distribution of modules.

The remaining three top-level modules are smaller. Each comprises a more functionally specialized set of structures which is not amenable to meaningful subdivisional breakdown according to the modularity analysis. What we call the “visual” module comprises the primary and associative areas of the dominant, tectofugal visual pathway of the pigeon brain along with its descending projections. The “viscero-limbic” module includes structures of the caudal limbic nidopallium and subnuclei of the amygdala. Finally, the “auditory” module comprises the primary, associative, and premotor areas of the auditory pathway. Figures 5, 6 show how the components of the five top-level modules are distributed within the telencephalon.

Having established that the pigeon forebrain is a small-world network with two levels of modularity, its connectivity matrix was further analysed to determine whether any of its nodes could be classified as hubs. A hub node is one that is topologically central, suggesting that it is likely to play an especially significant role in mediating the flow of information within the network. Nodes were ranked according to betweenness centrality (Table 3, left), in-degree, and out-degree (Table 4). The top five nodes for betweenness centrality, namely AD, AI, APH, CDL, and NCL, are the only five also to feature in the top ten for both in- and out-degree, and were hence classified as hubs. All five nodes also have high participation coefficients (>0.35) (Table 3), warranting their further classification as connector hubs, that is to say, nodes that are likely carry much of the information passing between modules.

**Table 3:**
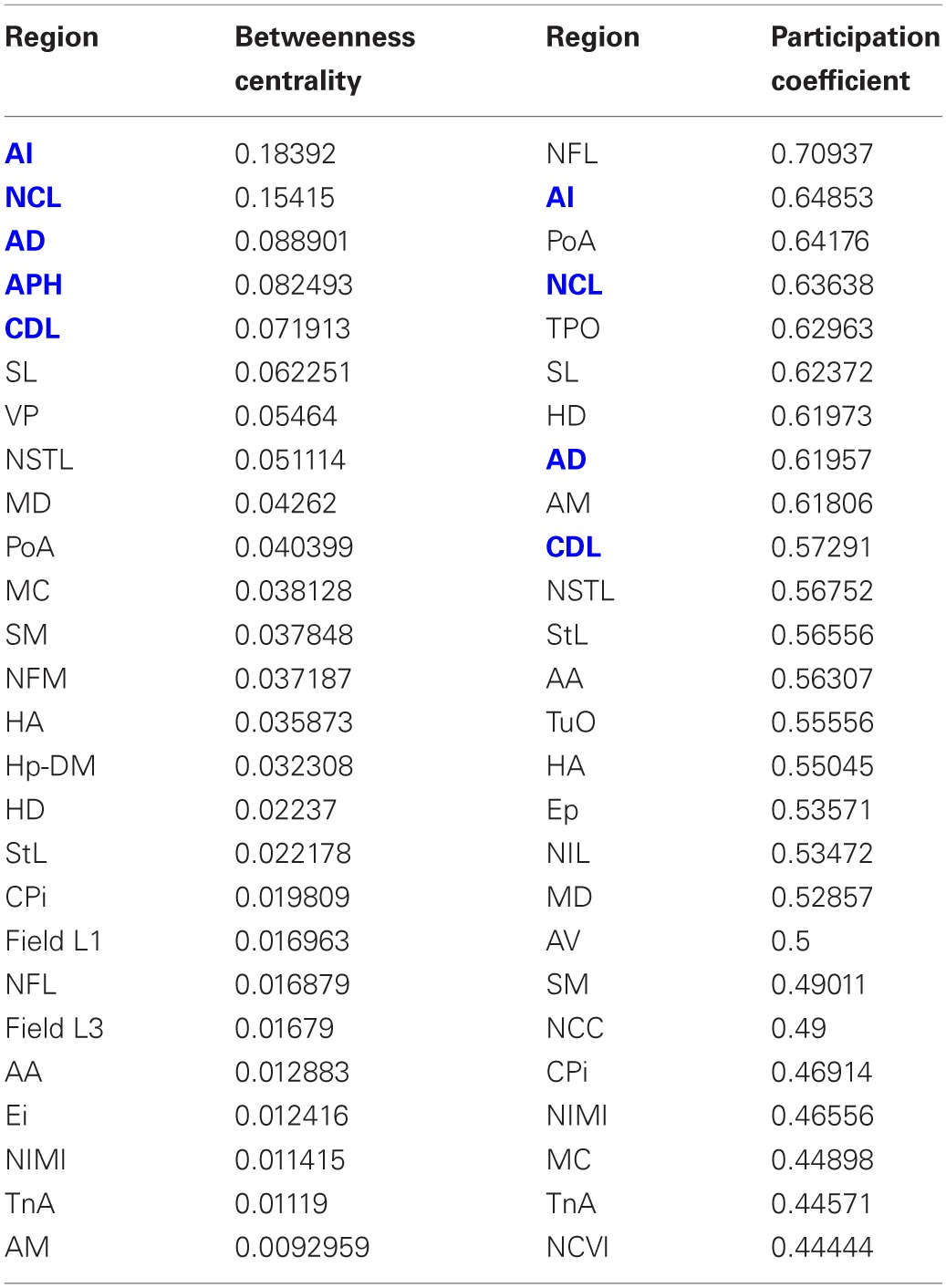
**Betweenness centrality and participation coefficients for the top 50% of the nodes in rank order**.

Connector hubs are highlighted. APH is ranked 29th for participation coefficient (so is not shown), with a value of 0.39639. Note that all nodes shown (as well as APH) have high participation coefficients. Values above 0.3 are conventionally taken to qualify a hub node for connector hub status.

**Table 4:**
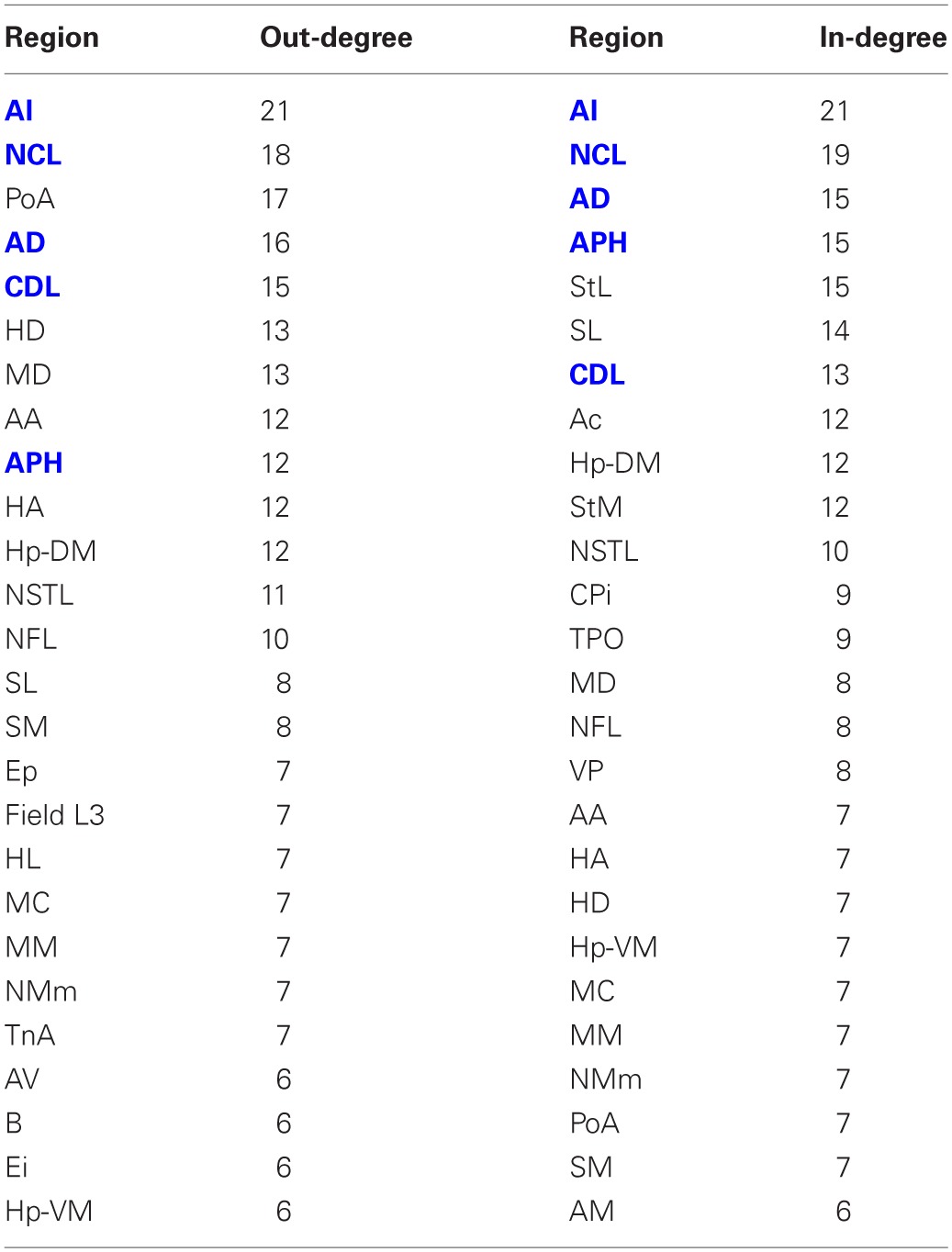
**Out-degree and in-degree for the top 50% of the nodes in rank order**.

Connector hubs are highlighted.

**Figure 7:**
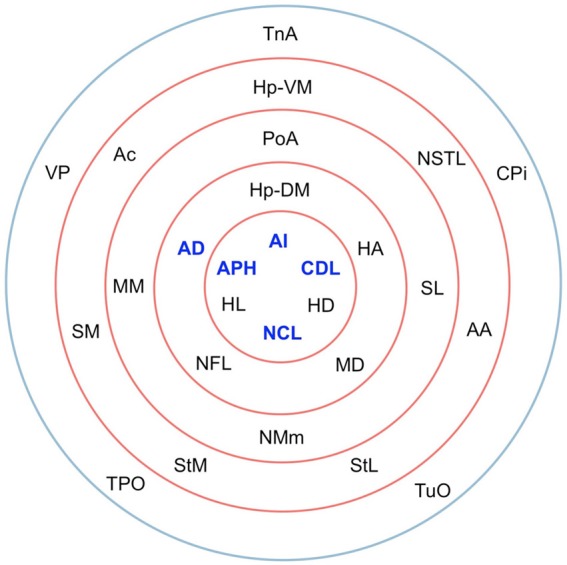
**Sub-shells of the innermost *k*-core following *k*-core decomposition**. The innermost *k*-core (*i* = 10) contains almost half the nodes in the network, but its sub-shell structure reveals a finer level of organization. All five hub nodes (shown in bold) appear in the innermost two sub-shells.

The matrix was also subjected to *k*-core decomposition (Table 5) using the algorithm described in the Methods (see Appendix). For the pigeon connectome, full erosion occurs at *i* = 11, and the innermost *k*-core (*i* = 10) contains just over half the nodes in the network. But when nodes are ranked according to sub-shell membership, four of the five connector hubs (AI, APH, CDL, and NCL) are seen to be in the innermost sub-shell, and all five connector hubs are among the 11 nodes in the innermost two sub-shells (Figure 7). Given their unique prominence according to all the network-theoretic measures used (node degree and betweenness centrality as well as *k*-core and sub-shell membership), the set of five connector hubs might be designated the connective core of the pigeon forebrain.

**Table 5:**
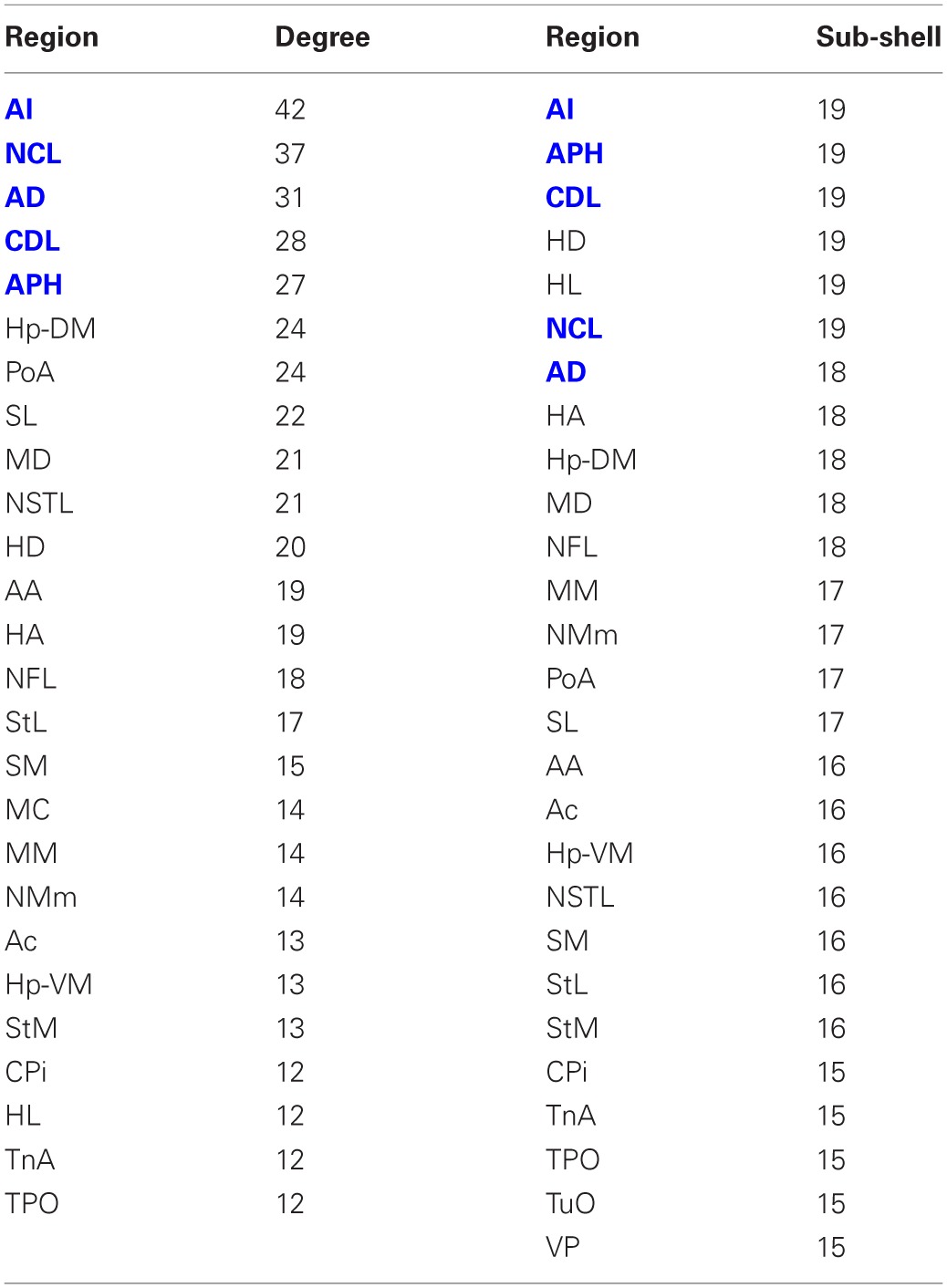
**Node degree and sub-shell number (following *k*-core decomposition) for the top 50% of the nodes in rank order**.

Connector hubs are highlighted. See also Figure 7.

**Figure 8:**
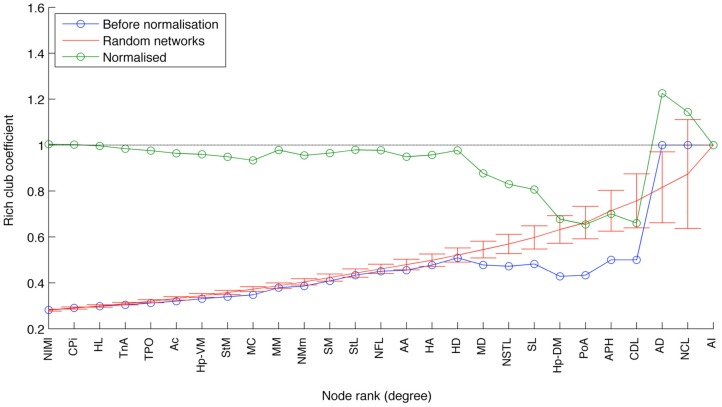
**The results of rich club analysis**. Nodes are ranked according to their total degree. The rich club coefficient for rank *k* is the proportion of possible connections between nodes of rank *k* or higher that are actual connections. This measure is then normalized with respect to the average for an equivalent random network. The three nodes at the rightmost end of the plot (AI, AD, and NCL) are designated a rich club, because their normalized rich club coefficients all lie above the random network average.

This designation gains qualified support from the application of two further measures that have proven useful for identifying the topologically central portions of the human and macaque brains, namely the rich club coefficient (van den Heuvel and Sporns, 2011; Harriger et al., 2012) and knotty-centrality (Shanahan and Wildie, 2012). A complex network possesses a rich club if a small subset of its nodes “own” a large proportion of its connectivity, and are also highly connected to each other. Analysis of the pigeon connectome reveals the presence of a rich club comprising three of the five connector hubs: AD, AI, and NCL (Figure 8). APH and CDL are excluded from the rich club because they lack sufficient connection with these three members. Finally, the knotty center of a complex network is a subset of its nodes that collectively “owns” a disproportionate amount of betweenness centrality as well as being highly intra-connected (Shanahan and Wildie, 2012). The knotty center of a brain network is likely to overlap with its rich club, if it has one. But the two concepts diverge in many cases. Analysis of the pigeon connectome yields a knotty center comprising four nodes: AD, AI, NCL, and MD (Mesopallium dorsal). In other words, the knotty center of the pigeon telencephalon contains all its rich club nodes, but also includes MD.

## Discussion

The present study shows that the pigeon's telencephalic connectome exhibits many of the network properties that have been found in mammals, such as a high small-world index, disassortativity, the prevalence of certain structural motifs, modularity, and the possession of a connective core of hub nodes. Although it has been claimed that low assortativity is a hallmark of a non-robust network, the opposite claim has been made for certain types of network (Zhou et al., 2012). So the implications of disassortativity in a brain network have yet to be understood. However, all the other network features can be broadly understood as supporting a balance of integration and segregation, a property thought to be essential for cognition in a large, distributed system of neurons (Sporns, 2013). A high small-world index is thought to permit efficient communication (integration) in a network without compromising its capacity for functional specialization (segregation) (Sporns and Zwi, 2004). Similarly, the combination of modules, connector hubs, and a connective core, as well as the structural motifs common to the pigeon and mammalian connectomes, have all been claimed to promote a combination of segregation and integration (Sporns and Kötter, 2004; Zamora-López et al., 2011; Shanahan, 2012; Sporns, 2013).

The pigeon connectome manifests two-levels of modularity with its top-level modules being functionally analogous to those of humans (see below). While the top-level modules of the human brain are anatomically localized, those of the pigeon brain are more anatomically distributed, so our topological analysis has revealed a pattern that is not necessarily manifest in spatial organization (Figures 5, 6). Moreover, the pigeon telencephalon has a topologically central connective core, indicated by multiple network measures, including betweenness centrality and node degree. The membership of this core is further supported by *k*-core decomposition, rich club analysis, and knotty centrality analysis. The hub nodes comprising the connective core are functionally analogous to hub nodes in the primate brain's topological core (see below). The presence of a topological core in birds as well as mammals adds further weight to the hypothesis that such connective infrastructure plays an important role in cognition (Zamora-López et al., 2011; Shanahan, 2012; Sporns, 2013).

### The modules of the pigeon telencephalon

The modularity analysis partitioned the network into five modules of which two could be further subdivided (Figure 4). We now briefly characterize these modules in anatomical and functional terms.

The associative module is constituted by two sub-modules of prefrontal and premotor nature.

The prefrontal sub-module incorporates all major associative areas of the pigeon brain that are linked to executive functions. These include NCL (a hub), which has been proposed as a functional analog of the mammalian prefrontal cortex based on hodological, electrophysiological, functional, and neurochemical evidence (Güntürkün, 2005). They also include NMm (Nidopallium mediale pars medialis), NIMl (Nidopallium intermedium mediale pars lateralis), and MM (Mesopallium mediale), regions that integrate input from all sensory streams and have reciprocal connections with the NCL (Kröner and Güntürkün, 1999; Atoji and Wild, 2012).

The premotor sub-module incorporates AD and AI, which are each hubs. Together with AA (Arcopallium anterior), these regions are considered premotor structures that innervate pallial, diencephalic, and brainstem structures down to cervical spinal levels (Zeier and Karten, 1971). At the same time AA, AD, and AI are associative structures that receive input from NCL (Leutgeb et al., 1996), auditory (Wild et al., 1993), trigeminal (Wild et al., 1985b), somatosensory (Kröner and Güntürkün, 1999), and visual structures (Bagnoli and Burkhalter, 1983; Husband and Shimizu, 1999).

The cortico-hippocampal module has two sub-modules (septo-hippocampal and limbic/olfactory) and integrates multimodal information that mediates, for instance, hippocampus-based spatial cognition.

The septo-hippocampal sub-module comprises hippocampal and limbic structures such as the parahippocampal area (APH), which has hub status. APH has been further subdivided (Atoji and Wild, 2004; Kahn and Bingman, 2009) and, together with ventromedial (Hp-VM) and dorsomedial hippocampus (Hp-DM), constitutes the core of the avian hippocampal formation (Atoji and Wild, 2006). These hippocampal components are interconnected with limbic structures: the medial (SM) and lateral (SL) septum, the diagonal band of Broca (NDB), and N. taeniae amygdalae (TnA) (Atoji and Wild, 2004). The avian hippocampal system is likely homologous to its mammalian counterpart (Reiner et al., 2004).

The limbic/olfactory sub-module is closely linked to the hippocampal formation and is composed of CDL, limbic-associated Wulst-subdivisions and olfactory structures. Output from HD (Hyperpallium dorsale) to the hippocampal formation may play a critical role in visually guided spatial memory (Kahn and Bingman, 2009). Pigeons also use olfactory cues for homing, and during homing the hippocampal formation, olfactory bulb (BO), and Cortex piriformis (CPi) are activated (Shimizu et al., 2004; Patzke et al., 2010). Interference with the hippocampus or olfactory structures results in disruptions in pigeon homing performance (Papi and Casini, 1990; Bingman et al., 2005; Gagliardo et al., 2011). Additionally, the BO has direct projections to TnA, which is connected to APH (Patzke et al., 2011).

The visual module represents tectofugal forebrain areas, which constitute the dominant visual system in pigeons. Entopallial subdivisions [Ee (Entopallium externum), Ei (Entopallium internum), and Ep (Entopallial belt)] are the origin of secondary projections to MVL (Mesopallium ventrolaterale), NIL (Nidopallium intermedium laterale), and TPO (Area temporoparietalis) (Husband and Shimizu, 1999; Krützfeldt and Wild, 2005). Ei also projects via the StL (lateral striatum) to Globus pallidus (GP) (Kuenzel et al., 2011). Thus, the visual module is composed of primary, associative, and descending (motor) aspects of the tectofugal system.

The viscero-limbic module components constitute the core of the avian pallial limbic system. PoA (N. posterioris amygdopalii), SpA (Area subpallialis amygdalae), and possibly AM (Arcopallium mediale) are considered components of the avian amygdala(Atoji et al., 2006). Viscerosensory afferents from the nucleus of the solitary tract (NSTL) project to the parabrachial nucleus and the bed nucleus of the stria terminalis (Katz and Karten, 1983; Arends et al., 1988). The parabrachial nucleus projects to both PoA and NSTL (Atoji et al., 2006), and NSTL projects upon the dorsal vagal complex (Berk, 1987). The limbic Nidopallium caudocentrale (NCC) receives relatively weak inputs from sensory dorsal thalamic nuclei and projects via medial arcopallium to medial hypothalamus (Atoji and Wild, 2009).

The auditory module represents subdivisions of the primary auditory fields in the telencephalon (L1–L3) and their connections with secondary (MC, Mesopallium caudale), associative (NCL), and premotor (AIvm, Arcopallium intermedium pars ventromedialis) structures (Wild et al., 1993).

### Comparing the avian and mammalian connectomes

Most of the modules of the pigeon telencephalon are functionally and/or anatomically comparable to modules that are revealed when network analysis is carried out on human brains (Hagmann et al., 2008). Both the pigeon and human forebrain possess a module that incorporates prefrontal, premotor, and motor fields and thus links associative sensory with motor areas (Güntürkün, 2005; Hagmann et al., 2008). The pigeon visual module incorporates the dominant visual system of this species and is in this respect functionally similar to the human occipital visual module (Shimizu and Bowers, 1999). The viscero-limbic module of the pigeon resembles the human cingulate and paracentral module with respect to its integrative and limbic components (Atoji and Wild, 2005; Margulies et al., 2009). The cortico-hippocampal module of the pigeon also has a direct counterpart in humans, which includes areas of the hippocampal complex as well as diverse primary and associative sensory systems (Atoji and Wild, 2006). Only the auditory module, the smallest complex identified by our analysis, lacks an identifiable counterpart in the modular structure of the human brain.

The hubs identified in our analysis are closely related to the hub nodes of humans, macaques, and cats (Sporns et al., 2007; Gong et al., 2009; Modha and Singh, 2010). In the macaque, most hubs are located in frontal cortex and encompass multiple prefrontal and supplementary motor/premotor areas. In the cat, areas la, lg, cgp, and 35 form part of the fronto-limbic hub cluster (Zamora-López et al., 2010). In humans, the superior frontal cortices are major hubs and members of the “rich club,” a collection of high-degree nodes that are more densely connected among themselves than with nodes of lower degrees (van den Heuvel and Sporns, 2011). Similarly in the pigeon brain, three out of five hubs are in the associative module (AD, AI, and NCL) and are of a prefrontal or premotor nature (Wild et al., 1985a; Kröner and Güntürkün, 1999; Güntürkün, 2005). CDL is a further hub of the pigeon connectome and has a similar connectivity pattern to the cingulate cortex (Atoji and Wild, 2005), which is also a hub in the connectomes of humans, macaques, and cats (Sporns et al., 2007; Gong et al., 2009; Modha and Singh, 2010; Zamora-López et al., 2010). In humans and monkeys several areas within perirhinal and parahippocampal lobe systems have hub status (Zamora-López et al., 2010; van den Heuvel and Sporns, 2011), as does the pigeon APH. Thus, most of the pigeon's hub nodes are functionally equivalent to one or several hubs in the macaque or cat brain, and in the case of the hippocampal formation we have probable homology as well as functional equivalence. Similar parallels can be drawn with hub nodes that have been identified in the human brain, both in prefrontal cortex (Hagmann et al., 2008; Gong et al., 2009; van den Heuvel and Sporns, 2011) and the hippocampal formation (Iturria-Medina et al., 2008; van den Heuvel and Sporns, 2011).

## Conclusion

The graph-theoretical analysis presented here reveals a connective core of five inter-connected hub nodes in the pigeon forebrain. In graph-theoretical terms, these regions are the most topologically central and most richly connected to the rest of the network, and are thus central to information flow in the avian brain. These findings are suggestive of the possibility that the same set of regions is central to avian cognition. Several researchers have hypothesized that intelligence evolved convergently in birds and primates (Emery and Clayton, 2004; Güntürkün, 2005). Our data are compatible with this idea, but hint at a somewhat more complex picture. For regions like the hippocampal APH, homology with their mammalian counterpart is likely, and the similarity of hippocampal network organization between birds and mammals is therefore likely due to shared evolutionary history. But several key structures in the pigeon connectome, such as NCL, AD, and AI, are functionally analogous but probably not homologous to corresponding mammalian structures (Medina and Reiner, 2000; Güntürkün, 2005). In these cases, shared network topology may be the outcome of convergent evolution. It is noteworthy that in both mammals and birds, the topologically central regions are also cognitively significant. It may therefore be reasonably hypothesized that during the evolution of taxa with demonstrably high cognitive abilities, similar selective pressures were at work resulting in similar network architectures.

Overall, our analysis suggests that, despite the absence of cortical layers, the avian brain conforms to the same organizational principles as the mammalian brain on a deeper, network-topological level. Future work will no doubt produce further refinements to the underlying connectome data. However, we anticipate that the central findings of the present paper will remain valid, namely the modular, small-world network topology of the avian brain and the presence within it of a connective core of hub nodes that includes hippocampal and prefrontal-like structures.

### Conflict of interest statement

The authors declare that the research was conducted in the absence of any commercial or financial relationships that could be construed as a potential conflict of interest.

## Appendix

The following algorithm was used for *k*-core and sub-shell decomposition.

A:= the set of all nodes in the graph
I:= 1 % core number
J:= 1 % sub-shell number

**while** A not empty

   **repeat**

      Recompute node degree k_i_ for each node i
      Deletions = {i: 0 < k_i_ < I}

      **for** each i in Deletions
         Core(i):= I-1
         Shell(i):= J
         Remove node i from A
      **end**

      J:= J+1

   **until** Deletions empty

    I:= I+1

**end**
